# The role of regulatory necrosis in traumatic brain injury

**DOI:** 10.3389/fnmol.2022.1005422

**Published:** 2022-10-18

**Authors:** Zhenyu Nie, Liming Tan, Jie Niu, Bing Wang

**Affiliations:** Department of Neurosurgery, The Second Affiliated Hospital, University of South China, Hengyang, China

**Keywords:** traumatic brain injury (TBI), regulatory necrosis, ferroptosis, necroptosis, pyroptosis, parthanatos, cell death

## Abstract

Traumatic brain injury (TBI) is a major cause of death and disability in the population worldwide, of which key injury mechanism involving the death of nerve cells. Many recent studies have shown that regulatory necrosis is involved in the pathological process of TBI which includes necroptosis, pyroptosis, ferroptosis, parthanatos, and Cyclophilin D (CypD) mediated necrosis. Therefore, targeting the signaling pathways involved in regulatory necrosis may be an effective strategy to reduce the secondary injury after TBI. Meanwhile, drugs or genes are used as interference factors in various types of regulatory necrosis, so as to explore the potential treatment methods for the secondary injury after TBI. This review summarizes the current progress on regulatory necrosis in TBI.

## Introduction

Traumatic brain injury (TBI) is a common traumatic disease and a serious factor causing death and disability in adults worldwide. Each year, more than 27 million new TBI cases are diagnosed around the world, imposing a huge burden on society and families (Jiang et al., 2019; Ponsford et al., 2022). TBI is a relatively complex disease, which will lead to structural damage and functional defects through primary and secondary injury mechanisms. Secondary injury occurs after primary injury, resulting from a cascade of metabolic, cellular and molecular events, and will eventually lead to brain cell death, tissue damage and atrophy (Ng and Lee, 2019). However, the cellular pathophysiological changes occurring in brain after TBI are mainly based on four major factors, namely excitotoxicity, cytokines, reactive oxygen species (ROS), and cell death (Ladak et al., 2019). In recent years, more and more studies have shown that some cell death is regulated by a certain kind of mechanism called regulatory necrosis, including necroptosis, pyroptosis, ferroptosis, parthanatos, and Cyclophilin D (CypD) mediated cell necrosis (Galluzzi et al., 2018). The details of regulatory necrosis in TBI and its differences in various features are provided in Table 1.

**TABLE 1:** Main morphological features, key regulators, inducers, and inhibitors of necroptosis, pyroptosis, ferroptosis, parthanatos, and CYPD-dependent necrosis.

Regulated necrosis	Main morphological features	Key regulators	Inducers	Inhibitors
Necroptosis	Loss of cytoplasmic membrane integrity, secretion of DAMP; swelling of cell bodies and organelles, chromatin fragmentation, nucleus disintegration	RIPK1, RIPK3, RIP1, RIP3, and MLKL	TNF-α,Fas, TRAIL, IFN, TNFR, TLR, and z-VAD-fmk	Nec-1, CYLD, 2-BFI, A20, CHMP4B, Arc, Hydrogen-rich saline, Perampanel, and HT
Pyroptosis	Cell swelling, cell membrane pore formation, release of bubble-like protrusions; cell membrane rupture, release of cell contents, DNA breakage; chromatin condensation, intact nuclei	NLRP3, ASC, Caspase-1, AIM2, GSDMD, IL-1β, and IL-18	ATP, LPS, PRR, HMGB1, and HIF-1α	Ac-YVAD-CMK, Ac-FLTD-CMK, PGAM5, VX765, JC124, NEK7, 2-BFI, ACE2, Dexmedetomidine, Artesunate, Resveratrol, Rhein, CORM-3, H2, and Ghrelin
Ferroptosis	Cellular mitochondria shrink in size and become smaller, with increased membrane density and reduced cristae. Insignificant morphological changes in the nucleus	Fe, GPX4, ROS, GSH, P53, and SLC7A11	Erastin, Erastin derivatives, RSL3, Glutamate, PEBP1, and 15LO	Ferrostatin-1, Liproxstatin-1, Deferoxamine, Ferristatin II, Baicalein, Prokineticin-2, Polydatin, Ruxolitinib, Tetrandrine, Melatonin, and SIRT2
Parthanatos	Loss of cell membrane integrity, intranuclear chromatin condensation, DNA breakage, and production of large amounts of DNA fragments; irreversible Δψm dissipation, ATP and NADH depletion	PARP-1, PAR, AIF, and MIF	PAR polymer, AIFsol (soluble AIF)	PARP-1 inhibitors, DPQ, GPI 6150, PJ34, INO-1001, Ghrelin, TSG, OLA, and Iduna
CYPD-dependent necrosis	Mitochondrial membrane potential damage; mitochondrial swelling, mitochondrial matrix expansion; massive intracellular vacuoles, outer membrane rupture	CypD, p53, and mPTP	CypD, VDAC (anion channel)	CsA, NIM811, Resveratrol, SIRT1, and BDNF

TRAIL, TNF-related apoptosis-inducing ligand; IFN, interferon; TNFR1, TNF-receptor 1; TLR, toll-like receptors; A20, TNF-inducible protein 3; CHMP4B, multivesicular body protein 4b; Arc, activity-regulated cytoskeletal; Perampanel, an (AMPAR) antagonist; HT, hypothermia treatment.

Regulatory necrosis has been found in many diseases in central nervous system, such as traumatic brain injury, spinal cord injury, epilepsy, Alzheimer’s disease (AD), Parkinson’s disease (PD), stroke, etc. (Liu et al., 2015; Sekerdag et al., 2018; Weiland et al., 2019; Hu et al., 2020; Dionísio et al., 2021). In Alzheimer’s disease, pTau can cause neuronal death by inducing necroptosis (Dong et al., 2022), while introducing the gene of amyloid precursor protein (App) can enhance necroptosis (Pang et al., 2022). In PD, fibrillar alpha-synuclein promotes the activation of neurotoxic astrocytes through RIP kinase signaling pathway (Chou et al., 2021). This review focuses on the role and the current studies of regulatory necrosis in the secondary injury after TBI, which may provide new targets for the treatment of craniocerebral injury.

## The necroptosis involved in traumatic brain injury

Necroptosis is induced by the combination of related ligands with Tumor Necrosis Factor (TNF) family death domain receptors, pattern recognition receptors, and virus sensors. It is a regulated cell death mode independent of caspase activity, which is mediated by mixed lineage kinase domain-like protein (MLKL) by activating receptor interacting protein kinase 1 (RIPK1)/receptor interacting protein kinase 3 (RIPK3) (Galluzzi et al., 2018). The process of necroptosis is characterized by cell swelling and the loss of plasma membrane integrity (Holler et al., 2000; Cho et al., 2009; Murphy et al., 2013; Grootjans et al., 2017; Degterev et al., 2019).

Previous studies have reported that necroptosis is involved in TBI (Liu et al., 2015; Yuan et al., 2019). Necroptosis would occur after a controlled cortical impact (CCI) in mice. RIPK3 is highly expressed in the hippocampus of CCI-TBI mice. Knockout of RIPK3 gene can inhibit oxidative stress, inflammation and apoptosis after TBI through AMPK signaling pathway (Liu Z. et al., 2018). The mice with RIPK3 gene knockout and RIPK1-deficient improved cognitive function within 3 months after TBI, demonstrating that the loss of RIPK1/RIPK3 could prevent progressive neuronal death and improve cognitive memory function (Wehn et al., 2021). But Wu et al. (2021) noted that the knockout of RIPK3 and MLKL in CCI mice model indicates RIPK3 is a disease driver independent of necroptosis mechanisms, while MLKL and the drug therapy of necroptosis may have no clinical effect on the patients with cerebral contusion. In PD animal model, the knockout of RIPK3 and MLKL can reduce the degeneration of dopaminergic neuron, improving the motion performance of mice (Oñate et al., 2020). The contribution of necroptosis to TBI needs to be further confirmed.

As the role of necroptosis in TBI has already been well recognized, many relevant studies started their research on the mechanisms that affected RIPK1/RIPK3/MLKL. Recently, Carsten Culmsee et al. found that mice with the knockout of tumor-suppressor cylindromatosis (CYLD) gene have relieved nerve damage after TBI. As a key regulator of deubiquitinase, cell proliferation and inflammation, the down-regulation of CYLD can increase the ubiquitination of RIP1, inhibit the formation of RIPK1/RIPK3 complex, and reduce necroptosis to protect neuronal cells (Ganjam et al., 2018). The 2-benzofuranyl-imidazoline (2-BFI) is an effective analgesic. In recent studies, 2-BFI treatment could significantly improve the neurological dysfunction and brain edema after TBI, of which mechanism is to reduce the level of receptor interacting proteins (RIPK1), (RIPK3), and MLKL (Ni et al., 2019). Other studies have shown that TNF Alpha induced protein 3 (TNFAIP3, also known as A20) can inhibit the synthesis of protein complexes composed of RIPK1, RIPK3, and MLKL, and thus reducing necroptosis in TBI, while Nec-1 and melatonin can reduce necroptosis and inhibit HMGB1, RAGE and proinflammatory cell factors in an A20 dependent manner (Bao et al., 2019). When MLKL maps to the site of damaged membrane bubble, it will recruit transport complex III (ESCRT-III) component (Gong et al., 2017; Guo and Kaiser, 2017), including the charged multivesicular body protein 4b (CHMP4B), which can alleviate the cell membrane damage caused by p-MLKL and the necroptosis level of microglia to a great extent. The transcription factor FOXO1 enhances the transcription of CHMP4B by binding to the promoter region in microglia. Stable knockdown of FOXO1 can reduce the expression of CHMP4B, thereby increasing the level of necroptosis after microglia damage, and further reducing the pro-inflammatory effect of microglia while improving the recovery of neural function after TBI (Zhao et al., 2020). According to current studies, the immediate-early gene (IEG) encoding the protein activity-regulated cytoskeletal (Arc) is a brain-specific postsynaptic density (PSD) protein. Arc can reduce the traumatic injury (TNI) in cortical neurons by inhibiting necroptosis. The arc silencing can activate the metabotropic glutamate receptor-1 (mGluR1) -mediated ER stress-calcium overload pathway and the RIP1-dependent necroptosis (Chen et al., 2020). As a AMPAR antagonist, perampanel has recently been reported as a neuroprotective factor in hemorrhagic and ischemic stroke models, while Wang et al. found that perampanel can also act as a protective factor in the TBI-*in vitro* model, reducing RIPK1 and RIPK3 expression and subsequently alleviating necroptosis through the activation of Akt/GSK3β signaling (Chen et al., 2021b).

**FIGURE 1:**
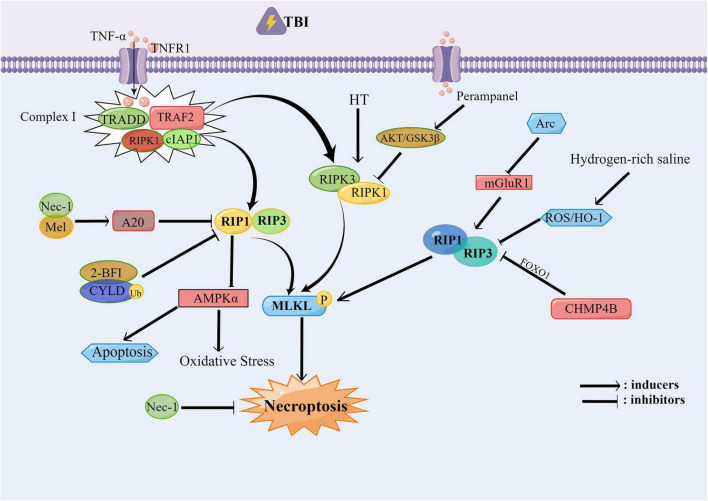
The necroptosis involved in traumatic brain injury (TBI). After TBI occurs, the RIPK1/RIPK3/MLKL pathway is activated, in which the Nec-1, Mel, 2-BFI, CYLD, and CHMP4B can alleviate the necroptosis and protect the neural function by inhibiting the RIP1 and RIP3. Hydrogen-rich saline can inhibit the RIP1 and RIP3 through the ROS/HO-1 pathway. While RIP1 can inhibit the oxidative stress and the apoptosis which occurred after TBI through the AMPK signal pathway. Perampanel can inhibit the RIPK1/RIPK3/MLKL pathway through the AKT/GSK3β pathway, thereby alleviating the necroptosis.

In fact, studies have shown that hydrogen or hydrogen-containing saline can modulate neuronal death. Hu et al. (2022) found that hydrogen-rich saline inhibits necroptosis and neuroinflammation based on the ROS/heme oxygenase-1 (HO-1) signaling, reducing neuronal death after TBI. As a research hotpot, Nec-1 is often used to verify the contribution of necroptosis. For example, Nec-1 can alleviate brain tissue injury, motor dysfunction and spatial learning impairment after CCI in mice, and has an anti-inflammatory effect in acute brain injury (You et al., 2008). Mu et al. (2021) found that Nec-1 can protect neuronal cells and oligodendrocytes by inhibiting the nuclear transposition of cellular AIF induced by the pro-apoptotic protein called Bcl-2/adenovirus E1B 19-kDa interacting protein 3 (BNIP3). At the same time, changes in external environment can also affect necroptosis. The hypothermia (HT) treatment can significantly reduce the upregulation of RIPK-1 and protect injured CNS from tissue damage and inflammation by targeting necroptosis through TNF signaling (Liu et al., 2016; Zhang et al., 2017) after TBI. The controlled decompression (CDC) surgery can reduce brain injury, and Chen et al. (2021a) stated that performing CDC for 2 or 3 h *in vitro* and for 20 or 30 min *in vivo* can exert neuroprotective effects. CDC can inhibit neuronal necroptosis through the TREK-1-mediated intracellular Ca^2+^ overload and the depression of RIPK3 activation. As indicated by Nec-1, necroptosis can affect acute neuronal injury, and the activation of RIPK1 and RIPK3 are both observed in the rat model of liquid impact brain injury and MCAO model with TBI (Liu et al., 2016; Ni et al., 2018). Interestingly, post-traumatic hypothermia (33°C) also reduces brain damage after stroke, resulting in decreased levels of RIPK1, RIPK3, and MLKL (You et al., 2008). Thus indicates that there may exist common target for the treatment of TBI and stroke by improving necroptosis. In conclusion, these studies have emphasized the potential therapeutic significance of necroptosis related therapy for TBI. The possible signal pathways of necroptosis involved in TBI are summarized in Figure 1.

## The pyroptosis involved in traumatic brain injury

Pyroptosis is mediated by Gasdermin D (GSDMD), the formation of plasma membrane pores as well as the extracellular release of inflammatory cytokines. In typical caspase-1 inflammatory pathway, caspase-1 is activated by apoptosis related CARD containing spotted protein (ASC) or pyridine domain 3 (NLRP3) in Nucleotide oligomerization domain (NOD) like receptor family, and processed into inflammatory cytokines such as IL-1β, IL-18 which can finally induce the release of inflammatory factors through the activation of GSDMD, resulting in cell death (Shi et al., 2017; Hu et al., 2020; Irrera et al., 2020; Zhou et al., 2022).

Therefore, most studies have been designed to explore the potential role of pyroptosis in TBI based on the regulation of inflammasome, such as caspase-1, NLRP1, NLRP3, AIM2, etc. Up to now, many of these studies have confirmed the contribution of pyroptosis to TBI by targeting inflammasomes. In the animal model of TBI, the caspase-1 plays a critical role, of which inhibition can reduce the level of IL-1β, IL-18, and GSDMD, and finally reduce the neuroinflammation and neuronal damage after TBI (Liu W. et al., 2018). Blocking the increasing level of phospho-Tau by IL-1R1^–/–^ in cortex and cerebellum suggests that inflammasome activation can drive Tau phosphorylation, while the aberrantly phosphorylated Tau may also contribute to neuronal IL-1β production and impaired proteostasis in feed forward loops, leading to neuronal death (Wu L. et al., 2022). The inflammasome plays a dominant role in the development of neuroinflammation after TBI, as NLRP3-GSDMD is dominant in the regulation of neuroinflammation and neuropathology after TBI. The level of GSDMD and N-GSDMD reach the peak 3 days after TBI, equivalent to the level of NLRP3 inflammasome. After TBI, GSDMD is mainly located in microglia cells, indicating that GSDMD may involve in the polarization of microglia cells. GSDMD-KO can alleviate the neuropathological changes (synaptic protein loss, microglia activation, astrocyte increase, dendritic damage and neuronal death) caused by TBI to a great extent (Du et al., 2022). The inhibition of GSDMD is conducive to a better prognosis, as the inhibition of inflammasome can prevent the neurological dysfunction in patients with TB1, PD, AD, subarachnoid hemorrhage, vascular dementia, etc. (Wnuk and Kajta, 2017; Ising et al., 2019; Rui et al., 2020; Poh et al., 2021). After TBI, NLRs, and AIM2 inflammatory corpuscles are activated in the cerebral microvascular endothelial cells (BMVECs) in cerebral cortex. As caspase-1 inhibitors, Ac-YVAD-CMK and Ac-FLTD-CMK can block the cleavage of GSDMD and ASC oligomerization by inhibiting caspase-1, which can reduce pyroptosis (Ge et al., 2018; Wang et al., 2021). Pgam5 is a mitochondrial protein that promotes the activation of microglial inflammasome after TBI, reduces the amount of pyroptosis-related molecules, promotes the polymerization of ASC and the activation of caspase1, and ameliorates the neuronal damage and dysfunction in TBI (Chen et al., 2021e). VX765, a known caspase-1 inhibitor, can inhibit pro-inflammatory cytokines against pyroptosis through HMGB1/TLR4/NF-κB pathway (Sun et al., 2020).

Pyridine domain 3 inflammasome is an intracellular multiprotein complex which can activate the release of inflammatory factors in TBI, causing cell pyroptosis (Irrera et al., 2017). Many researchers have found that NLRP3 inhibitors can inhibit cell death and play a neuroprotective role in TBI. JC124 is a specific NLRP3 inflammatory inhibitor, which is developed from the structural optimization of sulfonylurea drugs. It can block the aggregation of ASC, inhibit the activation of caspase-1 and protect brain from TBI (Kuwar et al., 2019). NIMA-associated kinase 7 (NEK7) is an important vector for NLRP3 inflammasome activation. Liu et al. have demonstrated that the NEK7 knockdown can inhibit the activation of NLRP3 inflammasome and caspase-1 through K^+^ outflow and reduce posttraumatic nerve injury (Chen et al., 2019). After TBI, the NLRP3 inflammasome inhibitor 2-BFI will induce inflammation and play an important role in BBB destruction and brain edema (Ni et al., 2019). Meanwhile, HIF-1α will recruit and activate microglia during the release of inflammatory factor, leading to the NLRP3 inflammasome-mediated cell pyroptosis (Yuan et al., 2021). Angiotensin converting enzyme 2 (ACE2) is an enzyme that catalyze the convert of angiotensin II to angiotensin, exerting neuroprotective effect. As proved by Meng Liang Zhou et al., ACE2 can reduce the mitogen activated protein kinase and NF in TBI- κ Phosphorylation of B, leading to the reduction of activated NLRP3 and caspase-3, thereby alleviating cell death (Li T. et al., 2022). As another effector molecule induced by the activation of NLRP3 inflammasome, high mobility group box 1 (HMGB1) is also involved in a typical damage-associated molecular pattern (DAMP), which is associated with the initiation process of NLRP3 inflammasome (Frank et al., 2015). Zhou et al. showed that NLRP3 inflammasome can impair the memory function in late TBI stages mainly through the upregulation of HMGB1 (Tan et al., 2021). Researchers have also studied some related drugs and found that dexmedetomidine, artesunate, and resveratrol can inhibit the activation of NLRP3 inflammasome, and thus presenting an anti-inflammatory function (Gugliandolo et al., 2018; Zheng et al., 2018; Zou et al., 2018).

**FIGURE 2:**
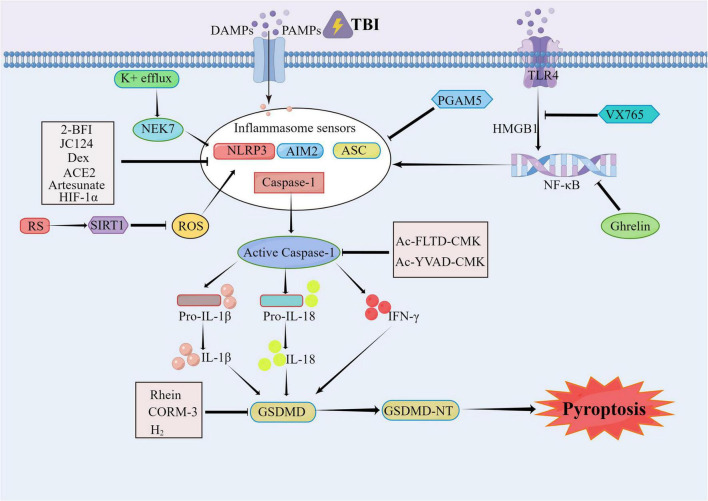
The pyroptosis involved in traumatic brain injury (TBI). After TBI occurs, the caspase-1 is mainly activated by NLRP3, AIM2, ASC, and other inflammasomes. The activated caspase-1 includes the IL-1β/18 lysed from the pro IL-1β, and the N-terminal segments of the GSDMD (GSDMD-NT) lysed from the Gasdermin D (GSDMD). The 2-BFI, JC124, Dex, ACE2, Artesunate, HIF-1α, and PGAM5 can alleviate the pyroptosis by inhibiting the inflammasomes. The VX765 can exert the neural protect function through the HMGB1/TLR4/NF-κB pathway. The Rhein, CORM-3, and H2 can inhibit the pyroptosis as well. The RS can activate the SIRT1 to inhibit the activation of NLRP3, thereby relieving the TBI. As the inhibitors of the Caspase-1, the Ac-YVAD-CMK and Ac-FLTD-CMK can inhibit the lysis of GSDMS and the oligomerization of ASC to alleviate the pyroptosis.

In addition, some natural products and gas molecules can also inhibit pyroptosis to improve the prognosis of brain injury such as Rhein, which protects the neurological dysfunction after TBI by inhibiting neuronal cell pyroptosis (Bi et al., 2020). Carbon monoxide releasing molecule-3 (CORM-3) is a water-soluble exogenous carbon monoxide involved in the two-way interaction between intestinal and brain, which can inhibit cell death and improve brain injury (Zhang et al., 2021). In terms of the complications after TBI, it has been reported that molecular hydrogen (H_2_) can improve the acute lung injury (ALI) after TBI in rats by reducing pyroptosis (Li T. T. et al., 2022). Meanwhile, as a neuroendocrine hormone and a new gastrointestinal hormone which can block NF-κB signaling pathway, ghrelin can improve the inflammasome induced focal necrosis and reduce the TBI induced ALI (Shao et al., 2020). Another study also showed that microglia and infiltrating CD11b^+^ leukocytes, which include macrophages and neutrophils, can actively participate in the innate immune response to penetrating brain injury (PBBI) and pyroptosis, which would lead to cell loss (Lee et al., 2019). These studies reported that pyroptosis signaling pathway might be a novel therapeutic target for TBI. The possible signal pathways of pyroptosis involved in TBI are summarized in Figure 2.

## The ferroptosis involved in traumatic brain injury

Ferroptosis is an iron dependent regulatory form of cell death driven by lipid peroxidation. It is characterized by the accumulation of intracellular iron and lipid ROS, the reduction of glutathione (GSH) level, and the inactivation of glutathione peroxidase 4 (GPX4) (Dixon et al., 2012; Stockwell et al., 2017; Yu et al., 2017; Chen et al., 2021d). Ferroptosis has been reported to be involved in TBI (Wenzel et al., 2017; Tang et al., 2020; Geng et al., 2021; Yao et al., 2021). Meanwhile, lipid peroxidation also plays an important role in the traumatic injury of nerve tissue (Anthonymuthu et al., 2018). In the TBI animal model, iron overload, the increased expression of transferrin, the accumulation of lipid ROS and mitochondrial atrophy associated with iron metabolic pathway further verified the existence of ferroptosis. While the treatment of ferroptosis with the inhibitor Fer-1 can reduce neuronal death and improve long-term cognitive and motor function (Xie et al., 2019). TfR1 is a recognized marker of ferroptosis. Researchers reported that ferristatin II (an iron absorption and TfR1 inhibitor) can inhibit the formation of ferritin by reducing Fe^3+^ and iron positive deposits, leading to the alleviation of the neuronal damage caused by TBI (Cheng et al., 2022).

In terms of the lipid metabolism pathway, some scholars have reported that in animal models of TBI, the expression levels of 15-HpETE-PE and 15LO2, GPX4 levels and enzyme activity are decreased in cerebral cortex and hippocampus, proving the existence of PEBP1/15LO-driven ferroptosis in TBI (Wenzel et al., 2017). Lipoxygenase (LOXs) is considered to be a key factor of ferroptosis. It inhibits 12/15-LOX while also reducing infarct size and improving behavioral parameters in ischemic stroke, which confirms the feasibility of 12/15-LOX inhibitors in the treatment of stroke (Karatas et al., 2018). It is reported that the redox lipomics method with liquid chromatography tandem mass spectrometry (LC-MS/MS) identify the oxidation of phosphatidylethanolamine (PEoX) and the reduction of glutathione levels. After the identification of PEoX as a predictive biomarker in ferroptosis by gas cluster ion beam secondary ion mass spectrometry (GCIB-SIMS) imaging and cluster ion beam, mapping the distribution of PEoX in cortical/hippocampal neurons after traumatic brain injury with a spatial resolution of 1.2 mm at single cell/subcellular level can help researchers visualize lipid peroxidation (Sparvero et al., 2021). At the same time, the baicalein administration (a 12/15-lipoxase inhibitor) can significantly reduce ferroptosis in TBI (Kenny et al., 2019). Moreover, baicalin also plays a neuroprotective effect against the seizures after TBI by inhibiting ferroptosis (Li et al., 2019). In addition, it is reported that prokineticin-2, as an important secretory protein, can participate in the pathogenesis of acute and chronic nervous system diseases. It reduces ferroptosis and protect nervous function through the ubiquitination of Fbxo10, the degradation of long chain acyl-CoA synthetase 4 (ACSL4) and the inhibition of lipid peroxidation (Bao et al., 2021).

In addition, there are some molecular compounds and drugs that involved in the mechanism of GPX4 inhibition that can cause ferroptosis. These compounds and drugs include polydatin, ruxolitinib, and tetrandrine. Among them, polydatin generally plays an anti-inflammatory effect, which can improve the activity of GPX4 enzyme and reduce MDA accumulation and lipid peroxidation deposition (Huang et al., 2021). As an inhibitor of janus kinase (JAK) 1 and 2, ruxolitinib is used to treat bone marrow fibrosis, which has an inhibitory effect on ferroptosis, and can also alleviate brain edema and nerve deformation (Chen et al., 2021c). Tetrandrine is a natural bisbenzylisoquinoline alkaloid that can ameliorate TBI by activating autophagy to reduce ferroptosis (Liu et al., 2022). Meanwhile, carotenoids can inhibit ferroptosis from I/R by increasing the expression of GPX4 (Guan et al., 2019). Selenium (Se) effectively inhibits GPX4-dependent ferroptosis, thereby protecting neurons and reducing cerebral infarction (Alim et al., 2019). Regulating the inhibition of ferroptosis by GPX4 may be an effective treatment for patients with ischemic stroke and TBI.

**FIGURE 3:**
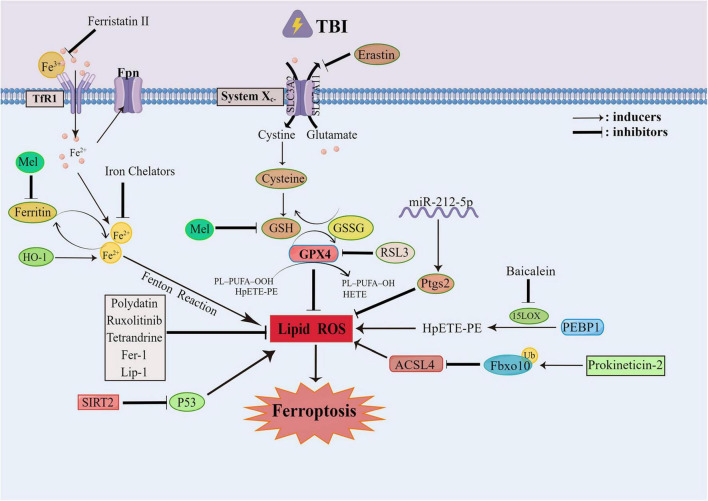
The ferroptosis involved in traumatic brain injury (TBI). The ferroptosis generally occurs after TBI, which can be targeted to improve the prognosis of TBI. The ferroptosis can be alleviated by the inhibition of the Xc-system and GPX4. The Mel can reduce the fenton reaction by inhibiting Ferritin. The Polydatin, Ruxolitinib, Tetrandrine can all alleviate the ferroptosis by mitigating the lipid peroxidation. While the SIRT2 can mitigate the lipid peroxidation through the inhibition of P53. The Prokineticin-2 can promote the ubiquitination of Fbxo10 to accelerate the degradation of Acsl4 and inhibit the lipid peroxidation. The miR-212-5p can inhibit the lipid peroxidation through Ptgs2. And the PEBP1/15LO can drive the occurrence of ferroptosis.

Some non-coding RNAs also exert function in the process of ferroptosis. The miR-212-5p can regulate Ptgs2 to inhibit ferroptosis and protect injured brain (Xiao et al., 2019). Melatonin is a neuroprotective factor which can mitigate lipid peroxidation through circPtpn14/miR-351-5p/5-LOX signaling. It can also antagonize ferroptosis and relieve ER stress in TBI (Wu C. et al., 2022). Referring to the protective mechanism of melatonin, Rui et al. (2021) found that melatonin could inhibit the neuronal FTH mediated ferroptosis after TBI. Meanwhile, p53 is another factor involved in ferroptosis, of which possible target is SLC7A11 (Jiang et al., 2015). Sirtuin 2 (SIRT2) is a member of nicotinamide adenine dinucleotide (NAD+) dependent protein deacetylase family which has neuroprotective effects on TBI by inhibiting the p53 mediated ferroptosis (Gao et al., 2021). To sum up, inhibiting ferroptosis can probably improve the damage caused by TBI. The possible signal pathways of ferroptosis involved in TBI are summarized in Figure 3.

## The parthanatos involved in traumatic brain injury

Parthanatos is a novel form of programmed cell death based on DNA damage and PARP-1 activation. In this process, the DNA repairment of poly ADP-PARP1 is over activated with the accumulation of intracellular poly ADP ribose (PAR) polymer, resulting in the depletion of NAD^+^ and ATP. PAR also combines with mitochondrial apoptosis which can induce the release of factor AIF to cell membrane. Combined macrophage migration inhibitory factor (MIF) can move to nucleus and split the genomic DNA into large fragments, causing chromatin condensation and fragmentation, and further leading to cell death (Virag and Szabó, 2002; Yu et al., 2006; Wang et al., 2011; Fatokun et al., 2014).

Multiple lines of evidence can support a certain role of parthanatos in TBI (Fatokun et al., 2014; Galluzzi et al., 2018). Secondary damage caused by oxidative stress after TBI will lead to DNA strand breakage, the over activation of PARP-1, and neuronal death. In some studies, the functional prognosis of TBI was improved by inactivation of PARP. This protective effect was confirmed by the use of a new PARP inhibitor named GPI 6150 (Virag and Szabó, 2002). PJ34 and INO-1001 are the other two structural PARP inhibitors except benzamide, which can reduce cell death and the microglia activation of primary cortical neurons exposed to *n*-methyl-*n*’-nitro-*N*-nitrosoguanidine (MNNG). They can also reduce reactive oxygen species neuroinflammation, and protect the neurons in cerebral cortex and thalamus. While neither of them can improve cognitive performance in morris water maze (MWM) test, nor can they reduce the loss of nerve cells in hippocampus (Stoica et al., 2014). INO-1001 can exert a neuroprotective effect in the rat TBI model by preventing NAD^+^ depletion (Besson et al., 2005; Clark et al., 2007). Meanwhile, the inhibition of NF-B-dependent gene transcription by PARP inhibition will prevent microglial activation. The inhibitor should be administered within 20 h after TBI, which will alleviate inflammation and improve histological and functional outcomes (d’Avila et al., 2012). Ghrelin also has the functions about improving sensormotor and reflex function and reducing cleaved PARP-1 levels in cortex, the PARP-1-dependent cell death, and the mortality after TBI (Qi et al., 2012). It is reported that tetrahydroxystilbene glucoside (TSG) is an active component of the traditional Chinese herbal medicine called polygonum multiflorum, which has neuroprotective effect. Its specific mechanism may be explicated as the reduction of oxidative stress and neuroinflammation and the inhibition of PARP1 to negatively regulate Ras/JNK signaling pathway (Cao et al., 2020). A large number of studies have focused on the pathway about transporting PARP inhibitors. Nanostructured lipid carriers (RBCNLCs) encapsulated by red blood cells (RBC) were used in brain neuron mitochondria together with C3 and ss31 peptides (C3/SS31-RBCNLCs). The high-concentration delivery of PARP inhibitor olapali (Ola) to brain mitochondria by C3/SS31-RBCNLCs-Ola has effectively improved mitochondrial function (Sun et al., 2022). Various lines of evidence suggested that the inhibition of PARP1 has a protective effect, as some studies found that PARP1 inhibition in ShRNA could promote axon regeneration, while the inhibition of other PARP isoforms would reduce axon regeneration with no improvement of motor function (Wang et al., 2016). The timing of pharmacological inhibition and the direction of inhibitor selection also need to be further investigated.

In addition to directly inhibiting PARP-1, aiming at PAR/MIF or NAD^+^ depletion pathway is also an option to improve the prognosis of TBI. It has been reported that the intranasal delivery of NAD^+^ can increase NAD^+^ levels in hippocampus and reduce the TBI induced hippocampal neuronal death (Won et al., 2012). Furthermore, MIF can mediate the TBI-induced neurodegeneration, neuronal death, and neurobehavioral dysfunction via its nuclease activity, while it shows no pro-inflammatory effects (Ruan et al., 2021). Recent studies demonstrated that iduna is a newly discovered ubiquitin E3 ligase and an endogenous regulator of parthanatos, which can reduce PARP activation and nuclear translocation of AIF to prevent parthanatos, indicating that ubiquitin-proteasome pathway may also play a role in parthanatos (Xu et al., 2019). At the same time, iduna may promote docosahexaenoic acid (DHA) through Wnt/MDM2 pathway and reduce the damage of TBI cell and mitochondrial dysfunction (Shi et al., 2022). Based on these reports, targeting PARP1-dependent parthanatos may be a potential strategy for the treatment of secondary injury after TBI.

## The cyclophilin D-mediated necrosis involved in traumatic brain injury

Cyclophilin D (CypD) is a member of cyclophilin family with various biological functions which can cause mitochondrial dysfunction through promoting the opening of mitochondrial permeability transitionpore (mPTP). For example, the loss of mitochondrial membrane potential, ATP depletion, mitochondrial swelling, and final mitochondrial outer membrane rupture can all induce the CypD pathway-dependent cell necrosis (Baines et al., 2005; Schinzel et al., 2005; Yamaguchi et al., 2005; Alam et al., 2015).

Evidence suggests that in the secondary damage generated after TBI, Cyclosporin A (CsA) can inhibit the opening of mPTP by interacting with CypD, resulting in the alleviation of mitochondrial dysfunction and neuronal damage in a TBI rat model (Sullivan et al., 2005; Kim et al., 2014; Springer et al., 2018). Studies have shown that the mice lacking CypD coding gene Ppif can retain mitochondrial function for 6 h after injury with fewer loss of subacute cortical tissue and hippocampal cells within 18 days after injury. As an effective inhibitor of CypD, CSA has many benefits about its usage on disease treatment (Readnower et al., 2021). There are many CSA related studies, some of them reported the function of CSA about suppressing mPTP opening that can maintain mitochondrial membrane potential and calcium balance in isolated mitochondria, and alleviate acute mitochondrial dysfunction after TBI (Sullivan et al., 1999). However, synaptic mitochondria will suffer more damage than non-synaptic mitochondria 24 h after CCI. While the intraperitoneal injection of CSA (20 mg/kg) at 15 mins after injury can improve synaptic and non-synaptic respiration to a significant extent, especially in the synaptic groups enduring more severe damage (Kulbe et al., 2017). As a non-immunosuppressive CSA analog, NIM811 as well as CSA can significantly reduce lipid peroxidation and protein nitrating damage of mitochondria 12 h after TBI. The neuroprotection provided by nim811 is dose-dependent with the most appropriate dose of 10 mg/kg. This dose can improve cognitive function and reduce mitochondrial damage (Mbye et al., 2008; Readnower et al., 2011). In preclinical experiments, positive improvements in brain metabolism and mitochondrial function were observed in TBI models in large animals, validating the neuroprotective effects of cyclosporine (Karlsson et al., 2019). At the same time, researchers have employed some research on the intervention of CypD. For example, CypD knockout can improve the abnormalities of excitatory synapses, while inhibiting CypD can reduce the synaptic overexcitation after TBI (Sun and Jacobs, 2016). But the knockdown of CypD was unable to reduce the pathology within axon initiation node (AIS), suggesting that axonal interval is regulated under different mechanism (Hanell et al., 2015).

Other studies have focused on the regulation of Cypd/mPTP in drugs or targeted molecules to improve mitochondrial function and produce protective effects. (1) Resveratrol can reduce mPTP opening by inhibiting the ROS mediated function, and protect the TBI mitochondrial function of GSK3 (Lin et al., 2014). (2) With an neuroprotective activity in p38 MAPK pathway, SIRT1 has been reported to protect mitochondria from damage (Yang et al., 2017). (3) Treatment of recombinant human erythropoietin or carbamylated erythropoietin can reduce mPTP opening caused by TBI, thereby improving mitochondrial disorders (Millet et al., 2016). (4) In rat brain mitochondria (RBM), the oxidative phosphorylation capacity (OXPHOS) can evaluate the respiratory effect of mitochondria. Etofoxine can restore mitochondrial oxidative phosphorylation and improve cognitive recovery after TBI (Palzur et al., 2021). (5) Brain-derived neurotrophic factor (BDNF) can inhibit the opening of MPTP, promote the accumulation of pCREB in mitochondrial intima and matrix and the synthesis of mitochondrial complex V, while alleviate the metabolic defects of neurons after mechanical injury (Xu et al., 2018). To sum up, the role of CypD-mediated necrosis in TBI can provide therapeutic implications for mitochondrial dysfunction after TBI.

## Discussion

Necroptosis, pyroptosis, ferroptosis, parthanatos, and CypD mediated necrosis are all important to the secondary injury after TBI. Several different types of regulatory necrosis can be triggered by nerve cells under death-inducing stimuli. However, under various complex pathophysiological mechanisms in TBI, these kinds of regulated necrosis may be interrelated and coexist with each other, or be alterable in cells with ever-changing levels. For example, (1) necroptosis may play a major role in the early stage after CCI, but other cell death pathways such as autophagy, apoptosis, pyroptosis, and ferroptosis are associated with the subsequent pathological process (Ganjam et al., 2018). (2) Silencing of RIPK1 can alleviate TBI by inhibiting inflammation and autophagy in neurons through NF-κB signaling pathway (Liu et al., 2020). (3) Nec-1 can prevent BNIP3 from integrating into mitochondria by modifying the binding site of BNIP3 on mitochondria. Therefore, Nec-1 can effectively inhibit the collapse of mitochondrial membrane potential induced by BNIP3 and reduce the opening efficiency of mPTP (Mu et al., 2021). Autophagy is significantly enhanced in TBI and ischemic stroke. The knockout of BNIP3 in mice can inhibit mitosis through the interaction of BNIP3 and LC3, with the manifestations of increased autophagy, decreased apoptosis and reduced cerebral infarction, indicating that the silencing of BNIP3 may be conducive to the neuroprotection after stroke (Shi et al., 2014). Meanwhile, Nec-1 can also inhibit the activation of necrotizing apoptosis as well as cell apoptosis and autophagy, while reducing the tissue damage and functional defects caused by TBI (Wang et al., 2012). (4) Autophagy activation can inhibit cell death in a mouse model of moderate traumatic brain injury through IL-13 and JAK1/STAT1 pathways (Gao et al., 2020). Inactivation of RIPK3 K51A kinase can enhance ferroptosis, causing worse outcomes after TBI. As a regulator of cell death, PEBP1 can inhibit the activity of pro-metabolic RIP3, and activate 15LOX to trigger pro-ferroptotic HpETE-PE signaling (Lamade et al., 2022). All these kinds of regulatory necrosis may occur simultaneously.

**FIGURE 4:**
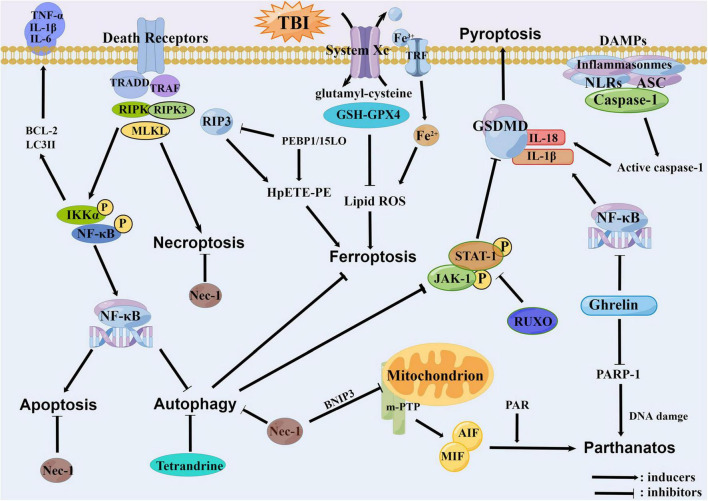
The relationships among all the regulatory necrosis involved in traumatic brain injury (TBI). The necroptosis can promote the apoptosis and inhibit the autophagy through the NF-κB pathway. The PEBP1 can interact with RIP3 or 15LOX to initiate the necroptosis and the ferroptosis. The Tetrandrine can inhibit the ferroptosis through the inhibition of autophagy. Meanwhile, the autophagy can inhibit the pyroptosis through the JAK1/STAT1 pathway. As an inhibitor of the necroptosis, the Nec-1 can concurrently inhibit the BNIP3 and decrease the open efficiency of mPTP, thereby reducing the parthanatos. The Ghrelin also has anti-inflammatory properties, which can alleviate the pyroptosis, and furthermore alleviate the parthanatos by reducing the PARP-1.

Interestingly, there are interactions between different types of cell death. Some reports showed that some inhibitors or hormones could be sensitive to another by blocking any way of cell death. For example, tetrahydropyrrole can improve TBI by regulating autophagy and reducing ferroptosis (Liu et al., 2022). It has been shown that treatment with 2-BFI could reduce both necroptosis and pyroptosis, thus exerting a role of neurofunctional protection (Ni et al., 2019). In some hormone treatments, ghrelin can reduce the level of cleaved PARP-1 in cortex, the PARP-1 dependent cell death and the mortality after TBI, while improving the sensorimotor and reflex functions. Its protective effect is related to its anti-inflammatory properties and pyroptosis (Shao et al., 2020). Upregulation of NIX reduces neuronal apoptosis and brain water content by increasing mitophagy in TBI rat model (Ma et al., 2019). Inhibition of autophagy and apoptosis and reduction of neuronal death using intranasal WNT3α therapy in TBI mice model can reduce the death of neurons (Zhang et al., 2018). In clinical stroke, Dl-3n-butylphthalide (Dl-NBP) has neuroprotective effects with anti-inflammatory, antioxidant, anti-apoptotic and mitochondrial protective functions. Dl-NBP treatment improves motor recovery after TBI by inhibiting the activation of autophagy and consequently blocking connexin loss and neuronal apoptosis (Wu et al., 2020). Therefore, regulatory necrosis may occur simultaneously with mutual transformation and interaction to some extent. The relationships among all the regulatory necrosis involved in TBI are summarized in Figure 4.

Secondary injury following TBI is a critical factor which affects prognosis. The cell death is an important cause of secondary injury and there is increasing number of researchers who have found that various regulatory necrosis could contribute to the development of TBI, providing many new perspectives for us to understand and treat TBI. Therefore, the intervention of regulatory necrosis related pathway may be an effective strategy to reduce the secondary injury after TBI, and the relationships among different necrosis are worthy of further study.

## Author contributions

ZN and BW: concept and design. ZN, LT, JN, and BW: writing, review, and revision of the manuscript. ZN: figures design. All authors approved the final version of the manuscript.

## References

[B1] Alam M. R. Baetz D. Ovize M. (2015). Cyclophilin D and myocardial ischemia-reperfusion injury: A fresh perspective. *J. Mol. Cell. Cardiol.* 78 80–89. 10.1016/j.yjmcc.2014.09.026 25281838

[B2] Alim I. Caulfield J. T. Chen Y. Swarup V. Geschwind D. H. Ivanova E. (2019). Selenium drives a transcriptional adaptive program to block ferroptosis and treat stroke. *Cell* 177 1262–1279. 10.1016/j.cell.2019.03.032 31056284

[B3] Anthonymuthu T. S. Kenny E. M. Lamade A. M. Kagan V. E. Bayır H. (2018). Oxidized phospholipid signaling in traumatic brain injury. *Free Radic Biol. Med.* 124 493–503. 10.1016/j.freeradbiomed.2018.06.031 29964171 PMC6098726

[B4] Baines C. P. Kaiser R. A. Purcell N. H. Blair N. S. Osinska H. Hambleton M. A. (2005). Loss of cyclophilin D reveals a critical role for mitochondrial permeability transition in cell death. *Nature* 434 658–662. 10.1038/nature03434 15800627

[B5] Bao Z. Fan L. Zhao L. Xu X. Liu Y. Chao H. (2019). Silencing of A20 aggravates neuronal death and inflammation after traumatic brain injury: A potential trigger of necroptosis. *Front. Mol. Neurosci.* 12:222. 10.3389/fnmol.2019.00222 31607859 PMC6761256

[B6] Bao Z. Liu Y. Chen B. Miao Z. Tu Y. Li C. (2021). Prokineticin-2 prevents neuronal cell deaths in a model of traumatic brain injury. *Nat. Commun.* 12:4220. 10.1038/s41467-021-24469-y 34244497 PMC8270965

[B7] Besson V. C. Zsengeller Z. Plotkine M. Szabo C. Marchand-Verrecchia C. (2005). Beneficial effects of PJ34 and INO-1001, two novel water-soluble poly(ADP-ribose) polymerase inhibitors, on the consequences of traumatic brain injury in rat. *Brain Res.* 1041 149–156. 10.1016/j.brainres.2005.01.096 15829224

[B8] Bi F. Ma H. Ji C. Chang C. Liu W. Xie K. (2020). Rhein protects against neurological deficits after traumatic brain injury in mice via inhibiting neuronal pyroptosis. *Front. Pharmacol.* 11:564367. 10.3389/fphar.2020.564367 33101024 PMC7554525

[B9] Cao Y. Chen Y. Wang F. Wang Y. Long J. (2020). PARP1 might enhance the therapeutic effect of tetrahydroxystilbene glucoside in traumatic brain injury via inhibition of Ras/JNK signalling pathway. *Folia Neuropathol.* 58 45–56. 10.5114/fn.2020.94006 32337957

[B10] Chen T. Yang L. Zhu J. Hang C. Wang Y. (2021b). The AMPAR antagonist perampanel regulates neuronal necroptosis via Akt/GSK3β signaling after acute traumatic injury in cortical neurons. *CNS Neurol. Disord. Drug Targets* 20 266–272. 10.2174/1871527319666201001110937 33001018

[B11] Chen T. Qian X. Zhu J. Yang L. K. Wang Y. H. (2021a). Controlled decompression attenuates compressive injury following traumatic brain injury via TREK-1-mediated inhibition of necroptosis and neuroinflammation. *Oxid. Med. Cell. Longev.* 2021:4280951. 10.1155/2021/4280951 34790287 PMC8592713

[B12] Chen Y. Gong K. Guo L. Zhang B. Chen S. Li Z. (2021e). Downregulation of phosphoglycerate mutase 5 improves microglial inflammasome activation after traumatic brain injury. *Cell Death Discov.* 7:290. 10.1038/s41420-021-00686-8 34642327 PMC8511105

[B13] Chen X. Kang R. Kroemer G. Tang D. (2021d). Broadening horizons: The role of ferroptosis in cancer. *Nat. Rev. Clin. Oncol.* 18 280–296. 10.1038/s41571-020-00462-0 33514910

[B14] Chen X. Gao C. Yan Y. Cheng Z. Chen G. Rui T. (2021c). Ruxolitinib exerts neuroprotection via repressing ferroptosis in a mouse model of traumatic brain injury. *Exp. Neurol.* 342:113762. 10.1016/j.expneurol.2021.113762 33991524

[B15] Chen T. Zhu J. Wang Y. Hang C. (2020). Arc silence aggravates traumatic neuronal injury via mGluR1-mediated ER stress and necroptosis. *Cell Death Dis.* 11:4. 10.1038/s41419-019-2198-5 31919348 PMC6952410

[B16] Chen Y. Meng J. Bi F. Li H. Chang C. Ji C. (2019). EK7 regulates NLRP3 inflammasome activation and neuroinflammation post-traumatic brain injury. *Front. Mol. Neurosci.* 12:202. 10.3389/fnmol.2019.00202 31555089 PMC6727020

[B17] Cheng Y. Qu W. Li J. Jia B. Song Y. Wang L. (2022). Ferristatin II, an iron uptake inhibitor, exerts neuroprotection against traumatic brain injury via suppressing ferroptosis. *ACS Chem. Neurosci.* 13 664–675. 10.1021/acschemneuro.1c00819 35143157

[B18] Cho Y. S. Challa S. Moquin D. Genga R. Ray T. D. Guildford M. (2009). Phosphorylation-driven assembly of the RIP1-RIP3 complex regulates programmed necrosis and virus-induced inflammation. *Cell* 137 1112–1123. 10.1016/j.cell.2009.05.037 19524513 PMC2727676

[B19] Chou T. W. Chang N. P. Krishnagiri M. Patel A. P. Lindman M. Angel J. P. (2021). Fibrillar alpha-synuclein induces neurotoxic astrocyte activation via RIP kinase signaling and NF-kappaB. *Cell Death Dis.* 12:756. 10.1038/s41419-021-04049-0 34333522 PMC8325686

[B20] Clark R. S. Vagni V. A. Nathaniel P. D. Jenkins L. W. Dixon C. E. Szabo C. (2007). Local administration of the poly(ADP-ribose) polymerase inhibitor INO-1001 prevents NAD+ depletion and improves water maze performance after traumatic brain injury in mice. *J. Neurotrauma* 24 1399–1405. 10.1089/neu.2007.0305 17711401

[B21] d’Avila J. C. Lam T. I. Bingham D. Shi J. Won S. J. Kauppinen T. M. (2012). Microglial activation induced by brain trauma is suppressed by post-injury treatment with a PARP inhibitor. *J. Neuroinflammation* 9:31. 10.1186/1742-2094-9-31 22335939 PMC3298794

[B22] Degterev A. Ofengeim D. Yuan J. (2019). Targeting RIPK1 for the treatment of human diseases. *Proc. Natl. Acad. Sci. U.S.A.* 116 9714–9722. 10.1073/pnas.1901179116 31048504 PMC6525537

[B23] Dionísio P. A. Amaral J. D. Rodrigues C. M. P. (2021). Oxidative stress and regulated cell death in Parkinson’s disease. *Ageing Res. Rev.* 67:101263. 10.1016/j.arr.2021.101263 33540042

[B24] Dixon S. J. Lemberg K. M. Lamprecht M. R. Skouta R. Zaitsev E. M. Gleason C. E. (2012). Ferroptosis: An iron-dependent form of nonapoptotic cell death. *Cell* 149 1060–1072. 10.1016/j.cell.2012.03.042 22632970 PMC3367386

[B25] Dong Y. Yu H. Li X. Bian K. Zheng Y. Dai M. (2022). Hyperphosphorylated tau mediates neuronal death by inducing necroptosis and inflammation in Alzheimer’s disease. *J. Neuroinflammation* 19:205. 10.1186/s12974-022-02567-y 35971179 PMC9377071

[B26] Du H. Li C. H. Gao R. B. Cen X. Q. Li P. (2022). Ablation of GSDMD attenuates neurological deficits and neuropathological alterations after traumatic brain injury. *Front. Cell Neurosci.* 16:915969. 10.3389/fncel.2022.915969 35669106 PMC9164823

[B27] Fatokun A. A. Dawson V. L. Dawson T. M. (2014). Parthanatos: Mitochondrial-linked mechanisms and therapeutic opportunities. *Br. J. Pharmacol.* 171 2000–2016. 10.1111/bph.12416 24684389 PMC3976618

[B28] Frank M. G. Weber M. D. Watkins L. R. Maier S. F. (2015). Stress sounds the alarmin: The role of the danger-associated molecular pattern HMGB1 in stress-induced neuroinflammatory priming. *Brain Behav. Immun.* 48 1–7. 10.1016/j.bbi.2015.03.010 25816800 PMC4508196

[B29] Galluzzi L. Vitale I. Aaronson S. A. Abrams J. M. Adam D. Agostinis P. (2018). Molecular mechanisms of cell death: Recommendations of the Nomenclature Committee on Cell Death 2018. *Cell Death Differ.* 25 486–541. 10.1038/s41418-017-0012-4 29362479 PMC5864239

[B30] Ganjam G. K. Terpolilli N. A. Diemert S. Eisenbach I. Hoffmann L. Reuther C. (2018). Cylindromatosis mediates neuronal cell death in vitro and in vivo. *Cell Death Differ.* 25 1394–1407. 10.1038/s41418-017-0046-7 29352268 PMC6113218

[B31] Gao C. Yan Y. N. Chen G. Wang T. Luo C. Zhang M. (2020). Autophagy activation represses pyroptosis through the IL-13 and JAK1/STAT1 pathways in a mouse model of moderate traumatic brain injury. *ACS Chem. Neurosci.* 11 4231–4239. 10.1021/acschemneuro.0c00517 33170612

[B32] Gao J. Li Y. Song R. (2021). SIRT2 inhibition exacerbates p53-mediated ferroptosis in mice following experimental traumatic brain injury. *Neuroreport* 32 1001–1008. 10.1097/WNR.0000000000001679 34102645

[B33] Ge X. Li W. Huang S. Yin Z. Xu X. Chen F. (2018). The pathological role of NLRs and AIM2 inflammasome-mediated pyroptosis in damaged blood-brain barrier after traumatic brain injury. *Brain Res.* 1697 10–20. 10.1016/j.brainres.2018.06.008 29886252

[B34] Geng Z. Guo Z. Guo R. Ye R. Zhu W. Yan B. (2021). Ferroptosis and traumatic brain injury. *Brain Res. Bull.* 172 212–219. 10.1016/j.brainresbull.2021.04.023 33932492

[B35] Gong Y. N. Guy C. Olauson H. Becker J. U. Yang M. Fitzgerald P. (2017). ESCRT-III Acts downstream of MLKL to regulate necroptotic cell death and its consequences. *Cell* 169 286–300. 10.1016/j.cell.2017.03.020 28388412 PMC5443414

[B36] Grootjans S. Vanden B. T. Vandenabeele P. (2017). Initiation and execution mechanisms of necroptosis: An overview. *Cell Death Differ.* 24 1184–1195. 10.1038/cdd.2017.65 28498367 PMC5520172

[B37] Guan X. Li X. Yang X. Yan J. Shi P. Ba L. (2019). The neuroprotective effects of carvacrol on ischemia/reperfusion-induced hippocampal neuronal impairment by ferroptosis mitigation. *Life Sci.* 235:116795. 10.1016/j.lfs.2019.116795 31470002

[B38] Gugliandolo E. D’Amico R. Cordaro M. Fusco R. Siracusa R. Crupi R. (2018). Neuroprotective effect of artesunate in experimental model of traumatic brain injury. *Front. Neurol.* 9:590. 10.3389/fneur.2018.00590 30108544 PMC6079305

[B39] Guo H. Kaiser W. J. (2017). ESCRTing necroptosis. *Cell* 169 186–187. 10.1016/j.cell.2017.03.030 28388403

[B40] Hanell A. Greer J. E. McGinn M. J. Povlishock J. T. (2015). Traumatic brain injury-induced axonal phenotypes react differently to treatment. *Acta Neuropathol.* 129 317–332. 10.1007/s00401-014-1376-x 25528329

[B41] Holler N. Zaru R. Micheau O. Thome M. Attinger A. Valitutti S. (2000). Fas triggers an alternative, caspase-8-independent cell death pathway using the kinase RIP as effector molecule. *Nat. Immunol.* 1 489–495. 10.1038/82732 11101870

[B42] Hu X. Chen H. Xu H. Wu Y. Wu C. Jia C. (2020). Role of pyroptosis in traumatic brain and spinal cord injuries. *Int. J. Biol. Sci.* 16 2042–2050. 10.7150/ijbs.45467 32549752 PMC7294939

[B43] Hu Y. Feng X. Chen J. Wu Y. Shen L. (2022). Hydrogen-rich saline alleviates early brain injury through inhibition of necroptosis and neuroinflammation via the ROS/HO-1 signaling pathway after traumatic brain injury. *Exp. Ther. Med.* 23:126. 10.3892/etm.2021.11049 34970349 PMC8713175

[B44] Huang L. He S. Cai Q. Li F. Wang S. Tao K. (2021). Polydatin alleviates traumatic brain injury: Role of inhibiting ferroptosis. *Biochem. Biophys. Res. Commun.* 556 149–155. 10.1016/j.bbrc.2021.03.108 33839410

[B45] Irrera N. Pizzino G. Calo M. Pallio G. Mannino F. Fama F. (2017). Lack of the Nlrp3 inflammasome improves mice recovery following traumatic brain injury. *Front. Pharmacol.* 8:459. 10.3389/fphar.2017.00459 28769794 PMC5509758

[B46] Irrera N. Russo M. Pallio G. Bitto A. Mannino F. Minutoli L. (2020). The Role of NLRP3 inflammasome in the pathogenesis of traumatic brain injury. *Int. J. Mol. Sci.* 21:6204. 10.3390/ijms21176204 32867310 PMC7503761

[B47] Ising C. Venegas C. Zhang S. Scheiblich H. Schmidt S. V. Vieira-Saecker A. (2019). NLRP3 inflammasome activation drives tau pathology. *Nature* 575 669–673. 10.1038/s41586-019-1769-z 31748742 PMC7324015

[B48] Jiang J. Gao G. Feng J. Mao Q. Chen L. Yang X. (2019). Traumatic brain injury in China. *Lancet Neurol.* 18 286–295. 10.1016/S1474-4422(18)30469-1 30784557

[B49] Jiang L. Hickman J. H. Wang S. J. Gu W. (2015). Dynamic roles of p53-mediated metabolic activities in ROS-induced stress responses. *Cell Cycle* 14 2881–2885. 10.1080/15384101.2015.1068479 26218928 PMC4825584

[B50] Karatas H. Eun J. J. Lo E. H. van Leyen K. (2018). Inhibiting 12/15-lipoxygenase to treat acute stroke in permanent and tPA induced thrombolysis models. *Brain Res.* 1678 123–128. 10.1016/j.brainres.2017.10.024 29079502 PMC5714685

[B51] Karlsson M. Pukenas B. Chawla S. Ehinger J. K. Plyler R. Stolow M. (2019). Neuroprotective effects of cyclosporine in a porcine pre-clinical trial of focal traumatic brain injury. *J. Neurotrauma* 36 14–24. 10.1089/neu.2018.5706 29929438 PMC6306685

[B52] Kenny E. M. Fidan E. Yang Q. Anthonymuthu T. S. New L. A. Meyer E. A. (2019). Ferroptosis contributes to neuronal death and functional outcome after traumatic brain injury. *Crit. Care Med.* 47 410–418. 10.1097/CCM.0000000000003555 30531185 PMC6449247

[B53] Kim S. Y. Shim M. S. Kim K. Y. Weinreb R. N. Wheeler L. A. Ju W. K. (2014). Inhibition of cyclophilin D by cyclosporin A promotes retinal ganglion cell survival by preventing mitochondrial alteration in ischemic injury. *Cell Death Dis.* 5:e1105. 10.1038/cddis.2014.80 24603333 PMC3973219

[B54] Kulbe J. R. Hill R. L. Singh I. N. Wang J. A. Hall E. D. (2017). Synaptic mitochondria sustain more damage than non-synaptic mitochondria after traumatic brain injury and are protected by cyclosporine A. *J. Neurotrauma* 34 1291–1301. 10.1089/neu.2016.4628 27596283 PMC5385586

[B55] Kuwar R. Rolfe A. Di L. Xu H. He L. Jiang Y. (2019). A novel small molecular NLRP3 inflammasome inhibitor alleviates neuroinflammatory response following traumatic brain injury. *J. Neuroinflammation* 16:81. 10.1186/s12974-019-1471-y 30975164 PMC6458637

[B56] Ladak A. A. Enam S. A. Ibrahim M. T. (2019). A review of the molecular mechanisms of traumatic brain injury. *World Neurosurg.* 131 126–132. 10.1016/j.wneu.2019.07.039 31301445

[B57] Lamade A. M. Wu L. Dar H. H. Mentrup H. L. Shrivastava I. H. Epperly M. W. (2022). Inactivation of RIP3 kinase sensitizes to 15LOX/PEBP1-mediated ferroptotic death. *Redox Biol.* 50:102232. 10.1016/j.redox.2022.102232 35101798 PMC8804265

[B58] Lee S. W. de Rivero Vaccari J. P. Truettner J. S. Dietrich W. D. Keane R. W. (2019). The role of microglial inflammasome activation in pyroptotic cell death following penetrating traumatic brain injury. *J. Neuroinflammation* 16:27. 10.1186/s12974-019-1423-6 30736791 PMC6367831

[B59] Li Q. Li Q. Jia J. Sun Q. Zhou H. Jin W. (2019). Baicalein Exerts neuroprotective effects in FeCl3-induced posttraumatic epileptic seizures via suppressing ferroptosis. *Front. Pharmacol.* 10:638. 10.3389/fphar.2019.00638 31231224 PMC6568039

[B60] Li T. T. Sun T. Wang Y. Z. Wan Q. Li W. Z. Yang W. C. (2022). Molecular hydrogen alleviates lung injury after traumatic brain injury: Pyroptosis and apoptosis. *Eur. J. Pharmacol.* 914:174664. 10.1016/j.ejphar.2021.174664 34883075

[B61] Li T. Huang H. Y. Wang H. D. Gao C. C. Liang H. Deng C. L. (2022). Restoration of brain angiotensin-converting enzyme 2 alleviates neurological deficits after severe traumatic brain injury via mitigation of pyroptosis and apoptosis. *J. Neurotrauma* 39 423–434. 10.1089/neu.2021.0382 34861788

[B62] Lin C. J. Chen T. H. Yang L. Y. Shih C. M. (2014). Resveratrol protects astrocytes against traumatic brain injury through inhibiting apoptotic and autophagic cell death. *Cell Death Dis.* 5:e1147. 10.1038/cddis.2014.123 24675465 PMC3973229

[B63] Liu H. He S. Wang J. Li C. Liao Y. Zou Q. (2022). Tetrandrine ameliorates traumatic brain injury by regulating autophagy to reduce ferroptosis. *Neurochem. Res.* 47 1574–1587. 10.1007/s11064-022-03553-9 35266084

[B64] Liu J. Zhu Z. Wang L. Du J. Zhang B. Feng X. (2020). Functional suppression of Ripk1 blocks the NF-κB signaling pathway and induces neuron autophagy after traumatic brain injury. *Mol. Cell. Biochem.* 472 105–114. 10.1007/s11010-020-03789-5 32666312

[B65] Liu T. Bao Y. H. Wang Y. Jiang J. Y. (2015). The role of necroptosis in neurosurgical diseases. *Braz. J. Med. Biol. Res.* 48 292–298. 10.1590/1414-431X20144310 25714887 PMC4418358

[B66] Liu T. Zhao D. Cui H. Chen L. Bao Y. Wang Y. (2016). Therapeutic hypothermia attenuates tissue damage and cytokine expression after traumatic brain injury by inhibiting necroptosis in the rat. *Sci. Rep.* 6:24547. 10.1038/srep24547 27080932 PMC4832230

[B67] Liu W. Chen Y. Meng J. Wu M. Bi F. Chang C. (2018). Ablation of caspase-1 protects against TBI-induced pyroptosis *in vitro* and *in vivo*. *J. Neuroinflammation* 15:48. 10.1186/s12974-018-1083-y 29458437 PMC5817788

[B68] Liu Z. Chen Q. Chen Z. Tian D. Li M. Wang J. (2018). RIP3 deficiency protects against traumatic brain injury (TBI) through suppressing oxidative stress, inflammation and apoptosis: Dependent on AMPK pathway. *Biochem. Biophys. Res. Commun.* 499 112–119. 10.1016/j.bbrc.2018.02.150 29470982

[B69] Ma J. Ni H. Rui Q. Liu H. Jiang F. Gao R. (2019). Potential roles of NIX/BNIP3L pathway in rat traumatic brain injury. *Cell Transplant.* 28 585–595. 10.1177/0963689719840353 30961359 PMC7103607

[B70] Mbye L. H. Singh I. N. Sullivan P. G. Springer J. E. Hall E. D. (2008). Attenuation of acute mitochondrial dysfunction after traumatic brain injury in mice by NIM811, a non-immunosuppressive cyclosporin A analog. *Exp. Neurol.* 209 243–253. 10.1016/j.expneurol.2007.09.025 18022160

[B71] Millet A. Bouzat P. Trouve-Buisson T. Batandier C. Pernet-Gallay K. Gaide-Chevronnay L. (2016). Erythropoietin and Its derivates modulate mitochondrial dysfunction after diffuse traumatic brain injury. *J. Neurotrauma* 33 1625–1633. 10.1089/neu.2015.4160 26530102

[B72] Mu J. Weng J. Yang C. Guan T. Deng L. Li M. (2021). Necrostatin-1 prevents the proapoptotic protein Bcl-2/adenovirus E1B 19-kDa interacting protein 3 from integration into mitochondria. *J. Neurochem.* 156 929–942. 10.1111/jnc.14993 32112403

[B73] Murphy J. M. Czabotar P. E. Hildebrand J. M. Lucet I. S. Zhang J. Alvarez-Diaz S. (2013). The Pseudokinase MLKL mediates necroptosis via a molecular switch mechanism. *Immunity* 39 443–453. 10.1016/j.immuni.2013.06.018 24012422

[B74] Ng S. Y. Lee A. Y. W. (2019). Traumatic brain injuries: Pathophysiology and potential therapeutic targets. *Front. Cell. Neurosci.* 13:528. 10.3389/fncel.2019.00528 31827423 PMC6890857

[B75] Ni H. Rui Q. Lin X. Li D. Liu H. Chen G. (2019). 2-BFI provides neuroprotection against inflammation and necroptosis in a rat model of traumatic brain injury. *Front. Neurosci.* 13:674. 10.3389/fnins.2019.00674 31293382 PMC6606784

[B76] Ni Y. Gu W. W. Liu Z. H. Zhu Y. M. Rong J. G. Kent T. A. (2018). RIP1K Contributes to neuronal and astrocytic cell death in ischemic stroke via activating autophagic-lysosomal pathway. *Neuroscience* 371 60–74. 10.1016/j.neuroscience.2017.10.038 29102662

[B77] Oñate M. Catenaccio A. Salvadores N. Saquel C. Martinez A. Moreno-Gonzalez I. (2020). The necroptosis machinery mediates axonal degeneration in a model of Parkinson disease. *Cell Death Differ.* 27 1169–1185. 10.1038/s41418-019-0408-4 31591470 PMC7205895

[B78] Palzur E. Edelman D. Sakas R. Soustiel J. F. (2021). Etifoxine restores mitochondrial oxidative phosphorylation and improves cognitive recovery following traumatic brain injury. *Int. J. Mol. Sci.* 22:12881. 10.3390/ijms222312881 34884686 PMC8657969

[B79] Pang K. Jiang R. Zhang W. Yang Z. Li L. L. Shimozawa M. (2022). An App knock-in rat model for Alzheimer’s disease exhibiting Abeta and tau pathologies, neuronal death and cognitive impairments. *Cell Res.* 32 157–175. 10.1038/s41422-021-00582-x 34789895 PMC8807612

[B80] Poh L. Fann D. Y. Wong P. Lim H. M. Foo S. L. Kang S. W. (2021). AIM2 inflammasome mediates hallmark neuropathological alterations and cognitive impairment in a mouse model of vascular dementia. *Mol. Psychiatry* 26 4544–4560. 10.1038/s41380-020-00971-5 33299135

[B81] Ponsford J. Spitz G. Hicks A. J. (2022). Highlights in traumatic brain injury research in 2021. *Lancet Neurol.* 21 5–6. 10.1016/S1474-4422(21)00424-5 34942137

[B82] Qi L. Cui X. Dong W. Barrera R. Nicastro J. Coppa G. F. (2012). Ghrelin attenuates brain injury after traumatic brain injury and uncontrolled hemorrhagic shock in rats. *Mol. Med.* 18 186–193. 10.2119/molmed.2011.00390 22160303 PMC3320141

[B83] Readnower R. D. Hubbard W. B. Kalimon O. J. Geddes J. W. Sullivan P. G. (2021). Genetic approach to elucidate the role of cyclophilin d in traumatic brain injury pathology. *Cells* 10:199. 10.3390/cells10020199 33498273 PMC7909250

[B84] Readnower R. D. Pandya J. D. McEwen M. L. Pauly J. R. Springer J. E. Sullivan P. G. (2011). Post-injury administration of the mitochondrial permeability transition pore inhibitor, NIM811, is neuroprotective and improves cognition after traumatic brain injury in rats. *J. Neurotrauma* 28 1845–1853. 10.1089/neu.2011.1755 21875332 PMC3172877

[B85] Ruan Z. Lu Q. Wang J. E. Zhou M. Liu S. Zhang H. (2021). MIF promotes neurodegeneration and cell death via its nuclease activity following traumatic brain injury. *Cell. Mol. Life Sci.* 79:39. 10.1007/s00018-021-04037-9 34921640 PMC8741753

[B86] Rui T. Wang H. Li Q. Cheng Y. Gao Y. Fang X. (2021). Deletion of ferritin H in neurons counteracts the protective effect of melatonin against traumatic brain injury-induced ferroptosis. *J. Pineal Res.* 70:e12704. 10.1111/jpi.12704 33206394

[B87] Rui W. Li S. Xiao H. Xiao M. Shi J. (2020). Baicalein Attenuates neuroinflammation by inhibiting NLRP3/caspase-1/GSDMD Pathway in MPTP Induced mice model of Parkinson’s Disease. *Int. J. Neuropsychopharmacol.* 23 762–773. 10.1093/ijnp/pyaa060 32761175 PMC7745250

[B88] Schinzel A. C. Takeuchi O. Huang Z. Fisher J. K. Zhou Z. Rubens J. (2005). Cyclophilin D is a component of mitochondrial permeability transition and mediates neuronal cell death after focal cerebral ischemia. *Proc. Natl. Acad. Sci. U.S.A.* 102 12005–12010. 10.1073/pnas.0505294102 16103352 PMC1189333

[B89] Sekerdag E. Solaroglu I. Gursoy-Ozdemir Y. (2018). Cell death mechanisms in stroke and novel molecular and cellular treatment options. *Curr. Neuropharmacol.* 16 1396–1415. 10.2174/1570159X16666180302115544 29512465 PMC6251049

[B90] Shao X. F. Li B. Shen J. Wang Q. F. Chen S. S. Jiang X. C. (2020). Ghrelin alleviates traumatic brain injury-induced acute lung injury through pyroptosis/NF-kappaB pathway. *Int. Immunopharmacol.* 79:106175. 10.1016/j.intimp.2019.106175 31918060

[B91] Shi J. Gao W. Shao F. (2017). Pyroptosis: Gasdermin-mediated programmed necrotic cell death. *Trends Biochem. Sci.* 42 245–254. 10.1016/j.tibs.2016.10.004 27932073

[B92] Shi R. Y. Zhu S. H. Li V. Gibson S. B. Xu X. S. Kong J. M. (2014). BNIP3 interacting with LC3 triggers excessive mitophagy in delayed neuronal death in stroke. *CNS Neurosci. Ther.* 20 1045–1055. 10.1111/cns.12325 25230377 PMC6492992

[B93] Shi X. Yang F. Ge Z. Tang Z. Zhang K. Chen B. (2022). Iduna contributes to the therapeutic effect of DHA in a cell and mouse model of traumatic brain injury via Wnt/MDM2 pathway. *Folia Neuropathol.* 60:92. 10.5114/fn.2021.112567 35359149

[B94] Sparvero L. J. Tian H. Amoscato A. A. Sun W. Y. Anthonymuthu T. S. Tyurina Y. Y. (2021). Direct mapping of phospholipid ferroptotic death signals in cells and tissues by gas cluster ion beam secondary ion mass spectrometry (GCIB-SIMS). *Angew. Chem. Int. Ed. Engl.* 60 11784–11788. 10.1002/anie.202102001 33684237 PMC8243396

[B95] Springer J. Prajapati P. Sullivan P. (2018). Targeting the mitochondrial permeability transition pore in traumatic central nervous system injury. *Neural Regen. Res.* 13 1338–1341. 10.4103/1673-5374.235218 30106036 PMC6108215

[B96] Stockwell B. R. Friedmann A. J. Bayir H. Bush A. I. Conrad M. Dixon S. J. (2017). Ferroptosis: A regulated cell death nexus linking metabolism, redox biology, and disease. *Cell* 171 273–285. 10.1016/j.cell.2017.09.021 28985560 PMC5685180

[B97] Stoica B. A. Loane D. J. Zhao Z. Kabadi S. V. Hanscom M. Byrnes K. R. (2014). PARP-1 inhibition attenuates neuronal loss, microglia activation and neurological deficits after traumatic brain injury. *J. Neurotrauma* 31 758–772. 10.1089/neu.2013.3194 24476502 PMC3967421

[B98] Sullivan P. G. Rabchevsky A. G. Waldmeier P. C. Springer J. E. (2005). Mitochondrial permeability transition in CNS trauma: Cause or effect of neuronal cell death? *J. Neurosci. Res.* 79 231–239. 10.1002/jnr.20292 15573402

[B99] Sullivan P. G. Thompson M. B. Scheff S. W. (1999). Cyclosporin A attenuates acute mitochondrial dysfunction following traumatic brain injury. *Exp. Neurol.* 160 226–234. 10.1006/exnr.1999.7197 10630207

[B100] Sun J. Jacobs K. M. (2016). Knockout of cyclophilin-D provides partial amelioration of intrinsic and synaptic properties altered by mild traumatic brain injury. *Front. Syst. Neurosci.* 10:63. 10.3389/fnsys.2016.00063 27489538 PMC4951523

[B101] Sun J. Liu J. Gao C. Zheng J. Zhang J. Ding Y. (2022). Targeted delivery of PARP inhibitors to neuronal mitochondria via biomimetic engineered nanosystems in a mouse model of traumatic brain injury. *Acta Biomater.* 140 573–585. 10.1016/j.actbio.2021.12.023 34958970

[B102] Sun Z. Nyanzu M. Yang S. Zhu X. Wang K. Ru J. (2020). VX765 Attenuates pyroptosis and HMGB1/TLR4/NF-kappaB pathways to improve functional outcomes in TBI mice. *Oxid. Med. Cell. Longev.* 2020:7879629. 10.1155/2020/7879629 32377306 PMC7181015

[B103] Tan S. W. Zhao Y. Li P. Ning Y. L. Huang Z. Z. Yang N. (2021). HMGB1 mediates cognitive impairment caused by the NLRP3 inflammasome in the late stage of traumatic brain injury. *J. Neuroinflammation* 18:241. 10.1186/s12974-021-02274-0 34666797 PMC8527642

[B104] Tang S. Gao P. Chen H. Zhou X. Ou Y. He Y. (2020). The role of iron, its metabolism and ferroptosis in traumatic brain injury. *Front. Cell. Neurosci.* 14:590789. 10.3389/fncel.2020.590789 33100976 PMC7545318

[B105] Virag L. Szabó C. (2002). The therapeutic potential of poly(ADP-Ribose) polymerase inhibitors. *Pharmacol. Rev.* 54 375–429. 10.1124/pr.54.3.375 12223530

[B106] Wang P. Pan B. Tian J. Yang L. Chen Z. Yang L. (2021). Ac-FLTD-CMK inhibits pyroptosis and exerts neuroprotective effect in a mice model of traumatic brain injury. *Neuroreport* 32:188. 10.1097/WNR.0000000000001580 33470761

[B107] Wang X. Sekine Y. Byrne A. B. Cafferty W. B. Hammarlund M. Strittmatter S. M. (2016). Inhibition of Poly-ADP-ribosylation fails to increase axonal regeneration or improve functional recovery after adult mammalian CNS injury. *eNeuro* 3:ENEURO.0270–16.2016. 10.1523/ENEURO.0270-16.2016 28032120 PMC5187389

[B108] Wang Y. Q. Wang L. Zhang M. Y. Wang T. Bao H. J. Liu W. L. (2012). Necrostatin-1 suppresses autophagy and apoptosis in mice traumatic brain injury model. *Neurochem. Res.* 37 1849–1858. 10.1007/s11064-012-0791-4 22736198

[B109] Wang Y. Kim N. S. Haince J. F. Kang H. C. David K. K. Andrabi S. A. (2011). Poly(ADP-ribose) (PAR) binding to apoptosis-inducing factor is critical for PAR polymerase-1-dependent cell death (parthanatos). *Sci. Signal.* 4:a20. 10.1126/scisignal.2000902 21467298 PMC3086524

[B110] Wehn A. C. Khalin I. Duering M. Hellal F. Culmsee C. Vandenabeele P. (2021). RIPK1 or RIPK3 deletion prevents progressive neuronal cell death and improves memory function after traumatic brain injury. *Acta Neuropathol. Commun.* 9:138. 10.1186/s40478-021-01236-0 34404478 PMC8369637

[B111] Weiland A. Wang Y. Wu W. Lan X. Han X. Li Q. (2019). Ferroptosis and Its role in diverse brain diseases. *Mol. Neurobiol.* 56 4880–4893. 10.1007/s12035-018-1403-3 30406908 PMC6506411

[B112] Wenzel S. E. Tyurina Y. Y. Zhao J. St C. C. Dar H. H. Mao G. (2017). PEBP1 wardens ferroptosis by enabling lipoxygenase generation of lipid death signals. *Cell* 171 628–641. 10.1016/j.cell.2017.09.044 29053969 PMC5683852

[B113] Wnuk A. Kajta M. (2017). Steroid and xenobiotic receptor signalling in apoptosis and autophagy of the nervous system. *Int. J. Mol. Sci.* 18:2394. 10.3390/ijms18112394 29137141 PMC5713362

[B114] Won S. J. Choi B. Y. Yoo B. H. Sohn M. Ying W. Swanson R. A. (2012). Prevention of traumatic brain injury-induced neuron death by intranasal delivery of nicotinamide adenine dinucleotide. *J. Neurotrauma* 29 1401–1409. 10.1089/neu.2011.2228 22352983 PMC5972775

[B115] Wu C. Du M. Yu R. Cheng Y. Wu B. Fu J. (2022). A novel mechanism linking ferroptosis and endoplasmic reticulum stress via the circPtpn14/miR-351-5p/5-LOX signaling in melatonin-mediated treatment of traumatic brain injury. *Free Radic. Biol. Med.* 178 271–294. 10.1016/j.freeradbiomed.2021.12.007 34883251

[B116] Wu F. Xu K. Xu K. Teng C. Zhang M. Xia L. (2020). Dl-3n-butylphthalide improves traumatic brain injury recovery via inhibiting autophagy-induced blood-brain barrier disruption and cell apoptosis. *J. Cell. Mol. Med.* 24 1220–1232. 10.1111/jcmm.14691 31840938 PMC6991645

[B117] Wu L. Chung J. Y. Cao T. Jin G. Edmiston W. R. Hickman S. (2021). Genetic inhibition of RIPK3 ameliorates functional outcome in controlled cortical impact independent of necroptosis. *Cell Death Dis.* 12:1064. 10.1038/s41419-021-04333-z 34753914 PMC8578385

[B118] Wu L. Kalish B. T. Finander B. Cao T. Jin G. Yahya T. (2022). Repetitive mild closed head injury in adolescent mice is associated with impaired proteostasis, neuroinflammation, and tauopathy. *J. Neurosci.* 42 2418–2432. 10.1523/JNEUROSCI.0682-21.2021 35105673 PMC8944232

[B119] Xiao X. Jiang Y. Liang W. Wang Y. Cao S. Yan H. (2019). miR-212-5p attenuates ferroptotic neuronal death after traumatic brain injury by targeting Ptgs2. *Mol. Brain* 12:78. 10.1186/s13041-019-0501-0 31533781 PMC6749650

[B120] Xie B. S. Wang Y. Q. Lin Y. Mao Q. Feng J. F. Gao G. Y. (2019). Inhibition of ferroptosis attenuates tissue damage and improves long-term outcomes after traumatic brain injury in mice. *CNS Neurosci. Ther.* 25 465–475. 10.1111/cns.13069 30264934 PMC6488926

[B121] Xu H. Li X. Wu X. Yang Y. Dai S. Lei T. (2019). Iduna protects HT22cells by inhibiting parthanatos: The role of the p53-MDM2 pathway. *Exp. Cell. Res.* 384:111547. 10.1016/j.yexcr.2019.111547 31472117

[B122] Xu Z. Lv X. A. Dai Q. Lu M. Jin Z. (2018). Exogenous BDNF Increases Mitochondrial pCREB and alleviates neuronal metabolic defects following mechanical injury in a MPTP-dependent way. *Mol. Neurobiol.* 55 3499–3512. 10.1007/s12035-017-0576-5 28508150

[B123] Yamaguchi O. Watanabe T. Kubo T. Yamagata H. Shimizu S. Tsujimoto Y. (2005). Cyclophilin D-dependent mitochondrial permeability transition regulates some necrotic but not apoptotic cell death. *Nature* 434 652–658. 10.1038/nature03317 15800626

[B124] Yang H. Gu Z. Li L. Maegele M. Zhou B. Li F. (2017). SIRT1 plays a neuroprotective role in traumatic brain injury in rats via inhibiting the p38 MAPK pathway. *Acta Pharmacol. Sin.* 38 168–181. 10.1038/aps.2016.130 28017962 PMC5309757

[B125] Yao M. Liu T. Zhang L. Wang M. Yang Y. Gao J. (2021). Role of ferroptosis in neurological diseases. *Neurosci. Lett.* 747:135614. 10.1016/j.neulet.2020.135614 33485988

[B126] You Z. Savitz S. I. Yang J. Degterev A. Yuan J. Cuny G. D. (2008). Necrostatin-1 reduces histopathology and improves functional outcome after controlled cortical impact in mice. *J. Cereb. Blood Flow Metab.* 28 1564–1573. 10.1038/jcbfm.2008.44 18493258 PMC2831087

[B127] Yu H. Guo P. Xie X. Wang Y. Chen G. (2017). Ferroptosis, a new form of cell death, and its relationships with tumourous diseases. *J. Cell. Mol. Med.* 21 648–657. 10.1111/jcmm.13008 27860262 PMC5345622

[B128] Yu S. W. Andrabi S. A. Wang H. Kim N. S. Poirier G. G. Dawson T. M. (2006). Apoptosis-inducing factor mediates poly(ADP-ribose) (PAR) polymer-induced cell death. *Proc. Natl. Acad. Sci. U.S.A.* 103 18314–18319. 10.1073/pnas.0606528103 17116881 PMC1838748

[B129] Yuan D. Guan S. Wang Z. Ni H. Ding D. Xu W. (2021). HIF-1alpha aggravated traumatic brain injury by NLRP3 inflammasome-mediated pyroptosis and activation of microglia. *J. Chem. Neuroanat.* 116:101994. 10.1016/j.jchemneu.2021.101994 34166779

[B130] Yuan J. Amin P. Ofengeim D. (2019). Necroptosis and RIPK1-mediated neuroinflammation in CNS diseases. *Nat. Rev. Neurosci.* 20 19–33. 10.1038/s41583-018-0093-1 30467385 PMC6342007

[B131] Zhang H. B. Cheng S. X. Tu Y. Zhang S. Hou S. K. Yang Z. (2017). Protective effect of mild-induced hypothermia against moderate traumatic brain injury in rats involved in necroptotic and apoptotic pathways. *Brain Inj.* 31 406–415. 10.1080/02699052.2016.1225984 28140659

[B132] Zhang J. Y. Lee J. H. Gu X. Wei Z. Z. Harris M. J. Yu S. P. (2018). Intranasally Delivered Wnt3a improves functional recovery after traumatic brain injury by modulating autophagic, apoptotic, and regenerative pathways in the mouse brain. *J. Neurotrauma* 35 802–813. 10.1089/neu.2016.4871 29108471 PMC5831263

[B133] Zhang L. M. Zhang D. X. Zheng W. C. Hu J. S. Fu L. Li Y. (2021). CORM-3 exerts a neuroprotective effect in a rodent model of traumatic brain injury via the bidirectional gut-brain interactions. *Exp. Neurol.* 341:113683. 10.1016/j.expneurol.2021.113683 33711325

[B134] Zhao P. Li C. Chen B. Sun G. Chao H. Tu Y. (2020). Up-regulation of CHMP4B alleviates microglial necroptosis induced by traumatic brain injury. *J. Cell. Mol. Med.* 24 8466–8479. 10.1111/jcmm.15406 32585748 PMC7412706

[B135] Zheng B. Zhang S. Ying Y. Guo X. Li H. Xu L. (2018). Administration of Dexmedetomidine inhibited NLRP3 inflammasome and microglial cell activities in hippocampus of traumatic brain injury rats. *Biosci Rep.* 38:BSR20180892. 10.1042/BSR20180892 30232232 PMC6435454

[B136] Zhou C. Zheng J. Fan Y. Wu J. (2022). TI: NLRP3 inflammasome-dependent pyroptosis in CNS trauma: A potential therapeutic target. *Front. Cell. Dev. Biol.* 10:821225. 10.3389/fcell.2022.821225 35186932 PMC8847380

[B137] Zou P. Liu X. Li G. Wang Y. (2018). Resveratrol pretreatment attenuates traumatic brain injury in rats by suppressing NLRP3 inflammasome activation via SIRT1. *Mol. Med. Rep.* 17 3212–3217. 10.3892/mmr.2017.8241 29257276

